# Open Questions: We don’t really know anything, do we? Open questions in sensory biology

**DOI:** 10.1186/s12915-017-0385-3

**Published:** 2017-05-17

**Authors:** Sönke Johnsen

**Affiliations:** Biology Department, Duke University, Durham, NC USA

## Abstract

Senses connect organisms to both the world and to each other, yet there is much we don’t know about them. Using examples drawn primarily from the author’s subfield of vision research, this article discusses five major open questions.

## To what extent do senses and signals co-evolve?

A classic hypothesis of sensory biology is that signals and senses co-evolve; in other words, an organism’s sensory capabilities influence the evolution of intraspecific signals, and the evolution of these signals in turn influences the evolution of sensory capabilities. This can be extended to interspecific interactions—for example, red warning coloration on a snake may be of little use if the predator to be warned lacks the ability to discriminate red from other colors. This co-evolution seems an intuitive hypothesis, and it is obvious that a signal must be detectable to function. In addition, we have marvelous examples that show an intimate connection between sensory function and signals. For example, one species of stomatopod has the rare ability to discriminate the circular polarization of light [1]. Circularly polarized light itself is also quite rare, but can be produced when light is reflected from the tail of this particular stomatopod. However, in this case and nearly all others, we do not know in which way the influence traveled, and if it travels equally often in both directions or even in both directions at all. In my own informal survey, it appears that most sensory biologists, including myself, are more willing to accept that signals evolve to better be detected by sensory systems than that sensory systems evolve to better detect a given signal. This judgment appears based on the assumption that it is easier to change a body odor or the color of a skin patch than it is to change what can be smelled or what colors can be seen. However, direct evidence confirming or refuting this is lacking at present.

## How are senses of different modalities combined?

**Fig. 1.**
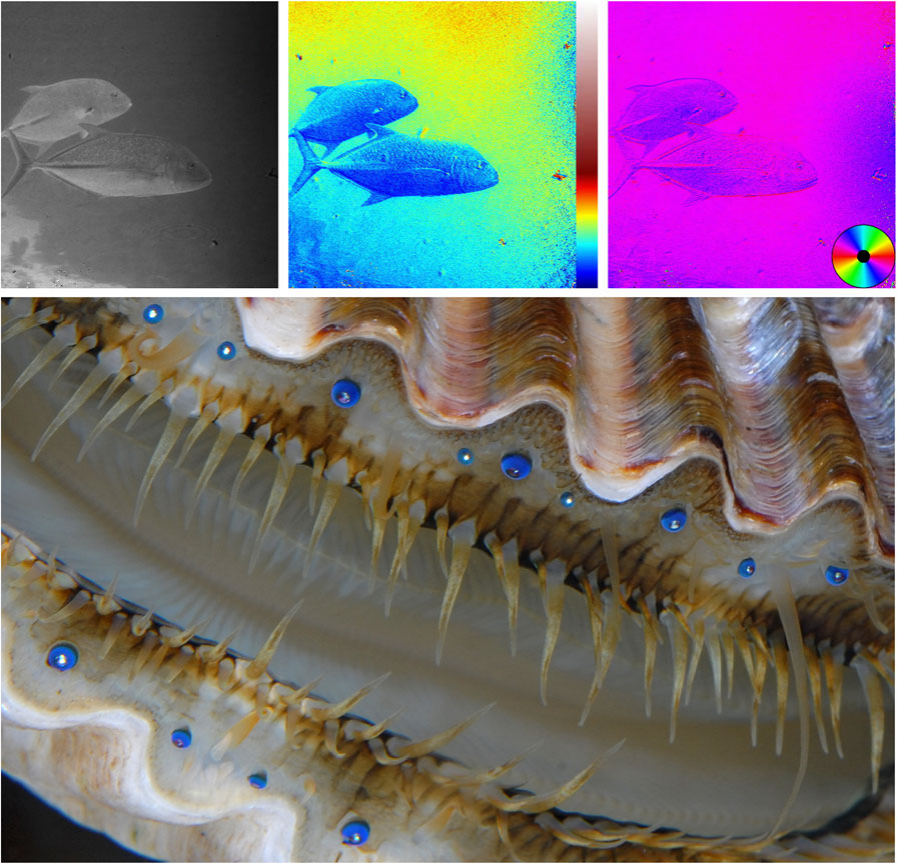
The top three images show (*left* to *right*) a normal image of the fish *Caranx melampygus*, an image of the degree of polarization of the image (with false-color scale to the *right*), and an image of the angle of polarization of the image (with false-color wheel scale in the *lower right*) (image courtesy of N. Justin Marshall). These three images show different aspects of the visual information contained in a scene, and it is not currently known if and how they are combined by animals with polarization vision. The bottom image is of the muliple mirror eyes of the bay scallop *Argopecten irradians*, which appear to collect far more information than the central nervous system of the animal can process (image courtesy of the author)

No organism is known to have only one sense, and many organisms, including humans, can be considered to have a few dozen senses, depending on how they are defined. Although it is possible that certain senses operate entirely in isolation, in general the information that an organism obtains is multimodal. For example, a billiard ball in one’s hand has weight, texture, temperature, and appearance. In addition, many modalities have sub-modalities. For example, the appearance of the billiard ball includes color, pattern, brightness, and—for those animals that can detect it—polarization information (i.e., the orientation of the light wave). Even within these sub-modalities, we are uncertain how the information is combined. In my own research field, there is considerable recent debate over how polarization, color, and brightness information are combined. For example, if a fish’s polarization and brightness contrast against the environment are both just below detectable limits, can the information be combined to render the animal detectable to a predator that can see both polarization and brightness (Fig. 1)? Similarly, how do brightness and hue combine to create our impression of color? These questions, while simple to ask, turn out to be difficult to answer. In addition, recent experiments show that additional sensory information may not always simply increase information content but actually change perceived information in another modality—for example seeing lip movements can affect the perception of speech (the McGurk Effect).

## What are the design principles and capabilities of distributed sensory systems?

Although humans have a number of distributed sensory systems, touch being the most obvious example, we tend to think of sensing as being accomplished via a small number of relatively large and complex organs (e.g., two eyes, two ears, one nose). This is not surprising; we depend heavily on our few complex sense organs, and organs of this type in other animals are relatively easy to find and thus more quickly discovered. However, this is a limited view. For example, chemoreception in many animals is not limited to the tongue and nose, and photoreception is not limited to eyes. Continuing with vision, even animals with discrete, complex eyes may have many more than two of them. Cubozoan jellies have 24 eyes, and scallops, chiton molluscs, and certain species of fan worms often have up to 100 [2] (Fig. 1). The eyes of scallops in particular are both numerous and complex, with each eye operating as a miniature Newtonian telescope, complete with corrective optics. In these molluscs and in cubozoa there is the baffling situation where the eyes appear to gather far more information than can be processed by their underdeveloped central nervous systems. Metaphorically, 100 television sets are on but nobody is watching. Even within vertebrates, we have fascinating distributed sensory systems such as the electroreceptive sense in weakly electric fish. This sense, despite having no known focusing structures, is able to discern the size, shape, and volume of distant objects using electric information gathered over much of the body [3]. This system, and distributed sensory systems in general, are constructed and operate via principles that we barely understand at all.

## What are the relative influences of phylogeny and physics on the evolution of sensory systems?

This question can be asked of any organismal trait, but is particularly interesting in the case of sensory systems because their architectures are so clearly influenced by physical principles. Consider a vertebrate eye versus a gene regulatory network. The former, so similar to cameras and so elegant in construction, is often given as proof of intelligent design. I am no expert on genomics, but have seen many charts of various gene networks, and would say that they, with their baroque complexities, apparent randomness, and redundancies, are as good a proof of the absence of intelligent design as one can get. The difference may be the apparently stronger influence of physics on the former. To be more precise, the binding of promoters to genes does of course depend on physics (as does everything), but physical principles do not appear to have an easily discernible influence on the structure of regulatory networks. However, due to the principles of optics, there are a limited number of ways to focus an image on a retina. If one wishes the eye to also work in low-light conditions, then the number of possible solutions is even more circumscribed. It is therefore perhaps not surprising that there are a relatively small number of eye designs, that convergent evolution is rampant, and that vision researchers are often accused of being adaptationists. This is not limited to vision or the macroscopic scale—for example, there are only so many fundamental types of mechanoreceptor cells. However, sense organs are not designed, and evolutionary history plays a role. The question is: to what extent? For example, a common question is whether arthropods are phylogenetically constrained to have compound eyes. This eye type, although it has its advantages (e.g., large field of view), has a number of disadvantages, particularly when it comes to the ability to resolve detail (a compound eye that sees as sharply as a human eye must be larger than a human head). Are there other advantages of compound eyes that we are unaware of, or are insects (for example) merely constrained by their phylogenetic history? Which senses are more bound by physics and which less? At first guess, one might assume that the macroscopic architecture of chemoreceptive systems is less constrained by physics than is vision, but even this is uncertain given the importance of fluid flow (both air and water) to the sense.

## How does magnetoreception work?

Hilbert’s famous “23 problems” in mathematics consisted primarily of general questions, but also included a few specific ones—Fermat’s Last Theorem, for example. Similarly, I end this article with a specific problem, that of the mechanism mediating magnetoreception. After some false starts, it is now well established via behavioral experiments that a large and phylogenetically diverse group of organisms can sense the strength and/or direction of the Earth’s magnetic field, often using it as a navigational cue [4]. However, with the exception of magnetotactic bacteria, which are arguably living compass needles, we are still not certain how this is accomplished. There are two major reasons for this. First, this is a not a sense that humans share, so we have no intuition as to where the sense organs might be or how they function. Second, magnetic fields, especially one as weak as the Earth’s, interact very little with nearly all matter and with biological tissue in particular. This has a number of consequences. To begin with, it is difficult to imagine a physical mechanism that can successfully detect the Earth’s field. Although electromagnetic induction has been considered in elasmobranchs, the two major hypotheses in other animals are: 1) tiny particles of iron oxide that affect channels or other cellular structures as they are rotated by the field, and 2) chemical reactions that are affected by magnetic fields. Both hypotheses have their advocates, but definitive evidence for either has proved to be difficult to obtain. In addition, because the interaction with tissue is so weak, the sense organ(s) could be anywhere inside the body, unlike eyes and ears, which have to be on the surface because the body would otherwise attenuate the stimulus. Finally, the weak interaction with the field implies that there are likely no larger accessory structures (e.g., outer ear, ocular lens) that focus and amplify the signal. This makes finding magnetoreceptors more difficult, and one is left searching for the metaphorical “needle in a needle stack”—what may be a microscopic, inconspicuous region of tissue surrounded by identically appearing tissue.

## Conclusion

As with all fields of knowledge, sensory biology has generated far more questions than answers, and the preceding is just a small sample generated by one person. Other questions raised by my colleagues include:

1. How much of perception is mediated by peripheral versus central mechanisms?
2. What factors cause a sensory system to evolve from general utility to high specificity?
3. How is sensory information represented in the central nervous system?
4. How does sensory perception within an individual depend on behavioral context?
5. How tightly linked are sensory and cognitive processes and can they in fact ever be considered separate?

As my father, a physicist, once put it, “biologists don’t really know anything, do they?” Unlike him though, I see this as wonderful and look forward to all that we will learn.
